# Local Glucocorticoid Administration Impairs Embryonic Wound Healing

**DOI:** 10.3390/biomedicines10123125

**Published:** 2022-12-03

**Authors:** Martin Bablok, Morris Gellisch, Beate Brand-Saberi, Gabriela Morosan-Puopolo

**Affiliations:** Department of Anatomy and Molecular Embryology, Institute of Anatomy, Medical Faculty, Ruhr University Bochum, 44801 Bochum, Germany

**Keywords:** wound healing, skin regeneration, mesenchymal cells, glucocorticoids, chicken embryo, bead-implantation, prenatal stress, fetal surgery

## Abstract

Understanding the complex processes of fetal wound healing and skin regeneration can help to improve fetal surgery. As part of the integumentary system, the skin protects the newborn organism against environmental factors and serves various functions. Glucocorticoids can enter the fetal circulatory system by either elevated maternal stress perception or through therapeutic administration and are known to affect adult skin composition and wound regeneration. In the present study, we aimed at investigating the effects of local glucocorticoid administration on the process of embryonic wound healing. We performed in-ovo bead implantation of dexamethasone beads into skin incisional wounds of avian embryos and observed the local effects of the glucocorticoid on the process of skin regeneration through histology, immunohistochemistry and in-situ hybridization, using vimentin, fibronectin, E-cadherin, Dermo-1 and phospho-Histone H3 as investigational markers. Local glucocorticoid administration decelerated the healing of the skin incisional wounds by impairing mesenchymal contraction and re-epithelialization resulting in morphological changes, such as increased epithelialization and disorganized matrix formation. The results contribute to a better understanding of scarless embryonic wound healing and how glucocorticoids might interfere with the underlying molecular processes, possibly indicating that glucocorticoid therapies in prenatal clinical practice should be carefully evaluated.

## 1. Introduction

With the emergence of fetal surgery in the last decades, various congenital malformations and pathologies such as diaphragmatic hernias, myelomeningocele, sacrococcygeal teratoma, and urinary tract obstructions are treatable today [1,2,3]. 

Fetal surgery aims at repairing developmental defects in utero, enabling the fetus to be delivered closer to term, ideally with restored organ function. One important requirement for successful fetal surgery is understanding the complex process of fetal wound healing and skin regeneration [4]. 

The restoration of a proper integumentary system is of high importance, because as the outermost barrier of an organism, the skin serves various life essential functions. It protects against environmental factors of physical or chemical nature but also plays a role in thermal regulation, vitamin synthesis, and exteroception [5]. 

Unlike adult wounds, which heal in three phases including inflammation, proliferation, and maturation [6], resulting in the formation of fibrotic tissue, fetal wounds are able to regenerate in a scarless manner [7]. The reason for that is the fact that fetal wound healing differs from adult wound healing in multiple ways. While in adult wounds, re-epithelialization of skin wounds is driven by means of lamellipodial crawling, embryonic wounds close by “actin purse-string contraction” [8,9]. For this, an actin cable composes at the epidermal wound margins within the first two minutes of injury in colocalization with myosin II, which enables the contractile machinery to implement [8,10]. For mesenchymal contraction, molecular differences have also been discovered. While in adult wounds the wound margins are drawn together by myofibroblasts, in fetal wounds these specialized cells do not intervene [10,11]. Fetal mesenchymal wound contraction is—as with the process of re-epithelialization—dependent on the actin cytoskeleton and strongly contributes to the total wound closure rate [11]. Moreover, the immune system plays an important role in the inflammatory processes of adult wounds. Due to the near absence of an active immune system during early development, inflammation does not occur in fetal wounds, possibly contributing to the unique capacity of non-fibrotic skin regeneration in fetal wounds [12].

Various intracellular and matrix proteins play an important role during skin repair and development. Fibronectin, for instance, has been described to act as a “substratum for cell migration” [13], mediating cell adhesion through cell surface proteins. For the intermediate filament protein vimentin, a lack of mesenchymal contraction has been described in wounded knock-out embryos [14]. As an epithelial adhesion marker, E-cadherin mediates wound closure through actomyosin remodeling during embryonic wound repair [15]. Focusing on skin development, Dermo-1 is known to be a relevant gene in skin and skin appendage establishment, as it is expressed in the subectodermal mesenchyme mediating dermal condensation [16,17]. 

A common pharmaceutical in prenatal medicine is dexamethasone (dex) as a synthetic glucocorticoid. It is mostly used to enhance prenatal fetal lung maturation in women at risk for preterm birth, reducing fetal mortality [18]. Besides the usage of synthetic glucocorticoids as pharmaceuticals, physiological equivalents such as maternal cortisol can pass the placental barrier for example due to maternal stress and affect fetal development in multiple ways [19,20,21,22]. Synthetic glucocorticoids show an increased affinity to the function-mediating glucocorticoid receptor in comparison to physiological glucocorticoids such as cortisol [23].

Unpublished investigations in our lab have shown, that prenatal systemic glucocorticoid-exposure impairs embryonic skin development. Focusing on the effect of glucocorticoids on the functionality of the skin, it has also been demonstrated that they can induce skin atrophy [24]. Moreover, it is well known that glucocorticoid-exposure impairs adult wound healing of the skin [25,26,27].

The effects of glucocorticoids on the process of fetal wound healing have not been thoroughly explored yet. Considering the high prevalence of glucocorticoid-exposure during fetal development as well as the requirement of adequate skin regeneration capacities for successful fetal surgery, we aimed at investigating the effects of local glucocorticoid administration on wound healing of the embryonic skin. 

To examine this research question, we used the chicken embryo as a model organism and applied dexamethasone locally into skin incisional wounds, employing the method of in-ovo bead implantation. The chicken embryo is a well-established model organism for prenatal stress and bead-implantation is commonly used by developmental biologists to analyze the effects of various substances on cellular actions during development [28,29,30,31,32].

Human fetal malformations caused by first-trimester systemic glucocorticoid administration, such as cleft lip as an example of cranial defects [33], can be reproduced and more thoroughly investigated in the avian embryo, employing molecular biological techniques [34]. 

Thus, our experimental setting of local dexamethasone-administration allows for a controlled and detailed exploration of glucocorticoid-induced changes on a cellular level, which are potentially applicable to clinical issues. 

Hypothesizing that local glucocorticoid administration impairs embryonic wound healing, we performed histological techniques, immunohistochemical methods, and in-situ hybridization to analyze the effects of dexamethasone on intracellular as well as extracellular matrix proteins, cellular proliferation, and further dermis development in wounded chicken embryos. 

## 2. Materials and Methods

### 2.1. Ethics Statement

According to German legislation, the use of embryonic vertebrates in an animal experiment needs approval only if the animal is in the last third of its embryonic development. In the case of chicken, this means that experiments performed on animals before embryonic day 14 are not regarded as an animal experiment by the German law and therefore, do not need approval or governmental permission.

### 2.2. Preparation of Dexamethasone-Beads

The beads were similarly prepared as previously described [32]. Briefly, the beads (AG 1-X2 resin, 143–1255, Bio-Rad, Hercules, CA, USA) were placed into a 0.4 mg/mL solution of dexamethasone (Sigma Aldrich, St. Louis, MO, USA) (dex) in phosphate-buffered saline (PBS) with 1% dimethyl sulfoxide (DMSO) overnight. The control beads were soaked in PBS with 1% DMSO. 

### 2.3. Chicken Embryo Treatment and In-Ovo Bead Implantation

Fertilized chicken eggs of *Gallus domesticus* were obtained from a local breeder, disinfected, and incubated at 37 °C and 80% humidity. The chicken embryos were staged according to Hamburger and Hamilton (HH) [35].

For our study, we used 70 fertilized chicken eggs, which were divided into a dexamethasone (dex) group (*n* = 35) and a control group (*n* = 35). 

On day 3 of incubation (Stages HH20-HH23), 3 mL of albumen was extracted with a syringe and a window was cut into the eggshell in order to visualize the embryo. Afterward, the window was sealed using medical tape. 

On day 4 of incubation (Stages HH23-HH24), we performed in-ovo bead implantation into skin incisional wounds. After visualizing the embryo by removing the medical tape, we partly opened the amnion covering the embryo, allowing access to the embryonic tissue. A skin incisional wound was made using a tungsten needle. The wounds were always made in the dorsolateral skin below the right forelimb. The dex- and control beads were inserted into the wound, using fine forceps. After the operation, the eggs were again covered with medical tape and further incubated. The process of wound healing was analyzed after 1 h and 2 days respectively. 

### 2.4. Histological Analysis

For histological analysis, fixed embryos were rinsed in PBS and dehydrated using ethanol. They were embedded in paraffin and sectioned transversally at 7 µm thickness. 

Paraffin sections were deparaffinized with RotiHistol (Carl Roth, Karlsruhe, Germany) and rehydrated for staining. 

For hematoxlin-eosin (HE) staining the sections were stained with hematoxylin for 15 min and eosin for 2 min (Carl Roth, Karlsruhe, Germany). 

Masson-Goldner-Trichrome-Staining was used in order to differentiate the connective tissue. First, the nuclei were stained for 5 min using hematoxylin according to Weigert. Then, the trichromatic stain was performed using ponceau-acid fuchsin, phosphotungstic acid-orange G, and 0.2% light green (Carl Roth, Karlsruhe, Germany). For differentiation, 1% acetic acid was used. 

After both staining procedures, the sections were dehydrated again and covered. The sections were analyzed microscopically and photographed using the virtual slide microscope VS120 (Olympus, Tokyo, Japan). Histological measurements and cell density calculation were performed with the OlyVia software (Version 2.9, Olympus, Tokyo, Japan) and QuPath (Open source software, Version 0.3.2). The beads were indicated by circles in order to improve visualization.

### 2.5. Immunohistochemical Analysis

Deparaffinized and rehydrated sections were microwaved in a citrate-buffer for unmasking of antigens, washed with PBS, permeabilized with 1% Triton X 100 (Sigma Aldrich, St. Louis, MO, USA), and blocked with 7.5% bovine serum in PBS.

Primary antibodies Vimentin (AMF-17b, DSHB), Fibronectin (B3/D6, DSHB) and phospho-Histone H3 (pHH3) (06-570, Merck, Darmstadt, Germany) were dissolved in the blocking solution and applied to the sections overnight.

After several washing steps the secondary fluorescent antibodies Alexa Fluor goat anti- mouse 488 and Alexa Fluor goat anti-mouse 568 (Thermo Fisher, Waltham, MA, USA) were put on the sections for an hour. The nuclei were stained with 4’,6-Diamidino-2-phenylindol (DAPI) (Carl Roth, Karlsruhe, Germany). 

B3/D6 was deposited to the DSHB by Fambrough, D.M. (DSHB Hybridoma Product B3/D6). AMF-17b was deposited to the DSHB by Fulton, A.B. (DSHB Hybridoma Product AMF-17b).

### 2.6. Whole Mount In-Situ Hybridization (ISH)

Whole mount in-situ hybridization was performed as previously described [36,37], using riboprobes for E-cadherin and Dermo-1. Permeabilization of the tissue was performed with proteinase K (10 μg/mL) for 10–40 min at room temperature, depending on the size of the embryo. Afterward, 1 μg/μL probe was hybridized for 48 h at 65 °C. The hybridized probe was visualized by an anti-DIG antibody conjugated to alkaline phosphatase (Sigma Aldrich, St. Louis, MO, USA).

Following ISH, the embryos were photographed with a stereo microscope (M165 FC, Leica, Wetzlar, Germany) equipped with a digital camera (DFC420 C, Leica, Wetzlar, Germany). The resulting photos were assembled in figures by using InkScape software (Open source software, Version 1.2.1, 2022).

For vibratome sectioning after in-situ hybridization, the embryos were embedded in 2.5 − 4% agarose gel and sectioned with a vibratome (VT 1000 S, Leica, Wetzlar, Germany) to 50μm. Sections were collected and covered with cover slips and Aquatex (Merck, Darmstadt, Germany). The sections were scanned using the AxioScan (Zeiss, Aalen, Germany) and edited with the microscopy software ZEN 3.6 (blue edition) (Zeiss, Aalen, Germany). 

### 2.7. Statistical Analysis

All data were tested for normality employing the Shapiro-Wilk test (*p* < 0.05). Afterward, the data were tested for significance using an unpaired t-test. Significance is declared by (*) for *p* < 0.05, (**) for *p* < 0.01, and (***) for *p* < 0.001. The results are presented as mean ± standard error. 

## 3. Results

### 3.1. Local Dexamethasone Administration Impairs Embryonic Wound Healing

In order to observe the effects of local dexamethasone (dex) administration on the regeneration process of embryonic skin incisional wounds, we performed in-ovo bead implantation into skin incisional wounds, with either dexamethasone-soaked beads or control beads. The regeneration process of dex wounds and control wounds was analyzed and compared after one hour and after two days, respectively. 

**Figure 1:**
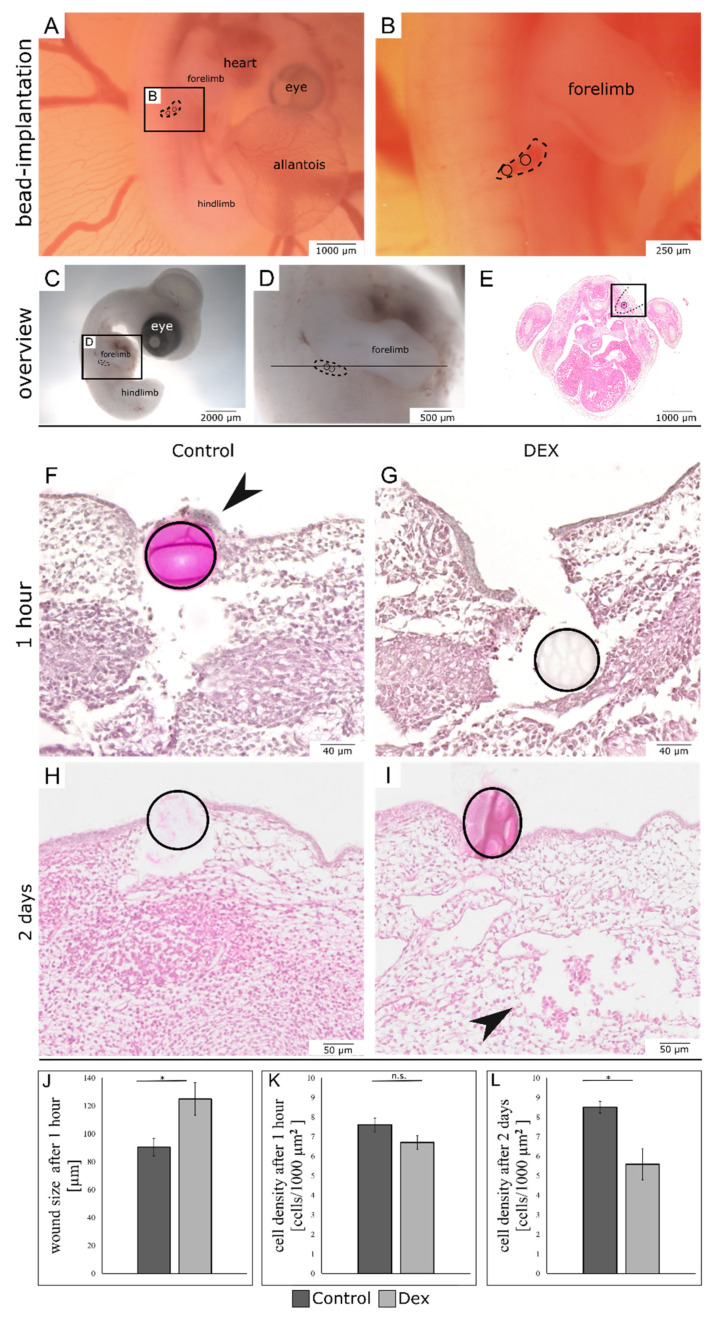
Overview of bead-implanted wounds. The images (**A**,**B**) display the method of in-ovo bead implantation in embryonic skin incisional wounds. The wound margins are indicated by dotted lines. (**C**,**D**) show macroscopic images of an exemplary embryo two days after wounding for orientation purposes. The line in (**D**) indicates the sectioning level. (**E**) shows an overview of an exemplary histological section, in which the wound area is indicated by a black square. (**F**) shows a control wound after one hour. (**G**) shows a dex wound after one hour. Note the impaired re-epithelialization in the dex wound in comparison to the regenerating epithelium in the control wound ((**F**), arrowhead). (**H**) shows a control wound after two days. (**I**) shows a dex wound after two days. Note the mesenchymal defects, blood infiltrations, and reduced cell density in the dex wound ((**I**), arrowhead). (**J**–**L**) show the quantification of different wound parameters. Data are presented as mean  ±  SE (control: *n* = 4; dex: *n* = 5 for both time points) * *p* < 0.05. *n*.s. = not significant.

For histological analysis, transverse sections of the bead-implanted areas (Figure 1C–E) were compared. After one hour the dex wounds showed a larger defect and the epithelium of the dex wound was restored to a lesser extent than in the control wounds (Figure 1F, arrowhead). Moreover, the subcutaneous mesenchyme of the dex wounds appeared less dense and disorganized in comparison to the control wounds. 

The wound size was quantified, as the distance between the two closing epithelial margins. The control wound size after one hour was 90.5 µm on average, while the wound size of dex wounds was 125 µm on average. This means, that after one hour the dex wounds were on average 38% significantly larger (*p* = 0.03; control: *n* = 4; dex: *n* = 5) (Figure 1J). 

For cell density quantification, the control wounds exhibited an average of 7.6 cells/1000 µm^2^, while the dex wounds exhibited an average of 6.7 cells/1000 µm^2^ after one hour (*p* = 0.07; control: *n* = 4; dex: *n* = 5) (Figure 1K).

**Figure 2:**
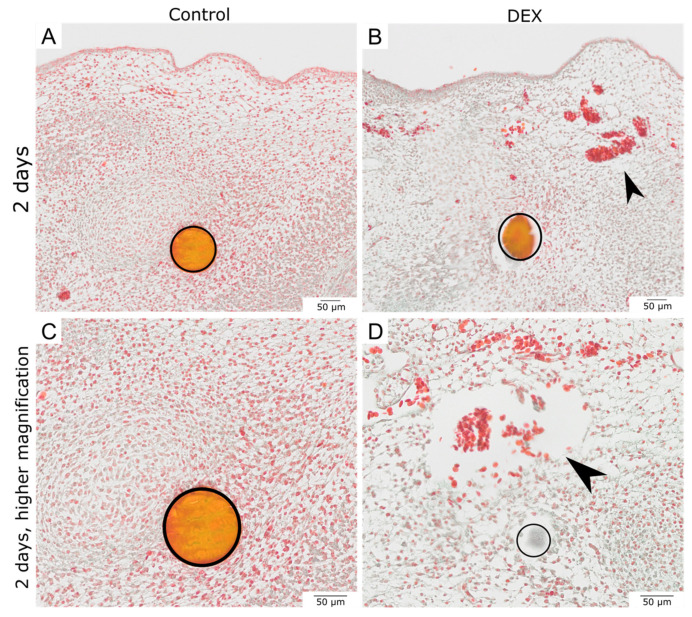
Masson-Goldner-Trichrome staining of bead-implanted wounds after two days. The images show control and dex wounds after two days in an overview (**A**,**B**) and in higher magnification (**C**,**D**). The Masson-Goldner-Trichrome-Staining visualizes the impairment of wound healing after two days caused by the dex beads. The subcutaneous hematoma in the mesenchyme appears prominently in the dex wounds (arrowheads).

After two days, both the dex- and control wounds had closed. The epithelium was restored in both experimental groups. While the subcutaneous mesenchyme of the control wounds showed a very dense accumulation of cells, especially in the area around the beads, the dex wounds showed defects in the subcutaneous mesenchyme (Figure 1I, arrowhead). The cell density was visibly reduced in the dex wounds. Moreover, the Masson-Trichrome-Goldner-staining revealed that after two days, the dex wounds developed a subcutaneous hematoma, presenting with extravascular erythrocytes (Figure 2B,D, arrowheads). The cell density in dex wounds was significantly reduced in comparison to control wounds after two days by 34.4% (*p* = 0.013; control: *n* = 4; dex: *n* = 5) (Figure 1L).

### 3.2. Local Dexamethasone Administration Reduces Vimentin Expression in Embryonic Wounds

**Figure 3:**
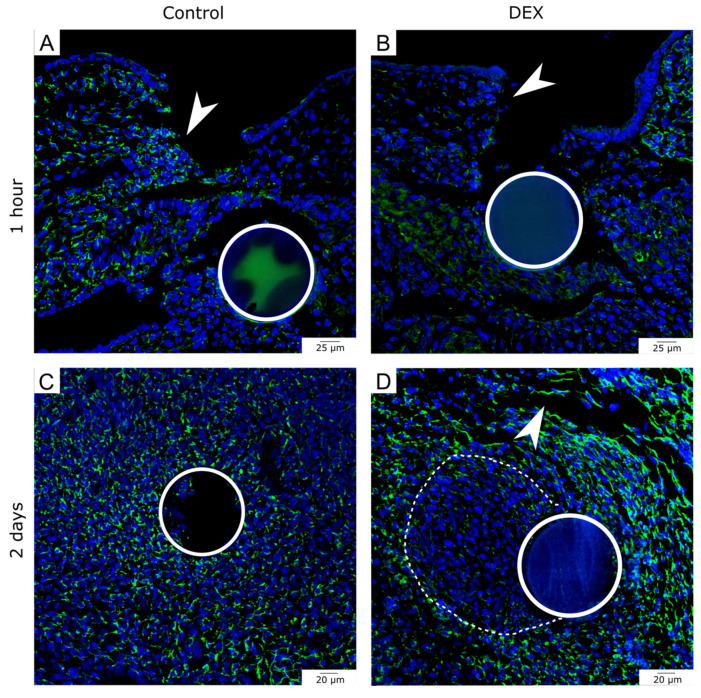
Vimentin expression in bead-implanted wounds. The images show immunohistochemistry for vimentin in the bead-implanted wounds. (**A**) shows a control wound after one hour. (**B**) shows a dex wound after one hour. Note the reduction of vimentin expression in the mesenchyme at the wound margin of the dex wound in comparison to the control (white arrows in (**A**,**B**)). (**C**) shows a control wound after two days. Note, that vimentin is detected up to the bead position. (**D**) shows a dex wound after two days. Note the disturbed expression pattern of vimentin in the dex wound. There is less expression in a radius around the bead (indicated by a dotted line in (**D**)), while there is strong expression around the mesenchymal defect (white arrowhead in (**D**)).

Immunohistochemical analysis revealed that after one hour vimentin expression was reduced in dex wounds. While the control wounds showed vimentin expression in the mesenchymal cells above the bead in the wound area (Figure 3A, arrowhead), the dex wounds showed a reduced expression of vimentin along the mesenchymal wound margins (Figure 3B, arrowhead). The reduction of vimentin expression in dex wounds in comparison to the control wounds after one hour was observed in 80% of the experimental group (control: *n* = 4; dex: *n* = 5). 

After two days, the control wound showed a dense subcutaneous mesenchyme with a uniform expression pattern of vimentin (Figure 3C). The dex wounds showed a disturbed and disorganized expression pattern. While along the mesenchymal defects, the expression of vimentin was prominent (Figure 3D, arrowhead), almost no expression of vimentin in the immediate radius around the dex bead occurred (Figure 3D, dotted line). The reduction of vimentin expression in dex wounds in comparison to the control wounds after two days was observed in 100% of the experimental group (control: *n* = 4; dex: *n* = 5). 

### 3.3. Local Dexamethasone Administration Reduces Fibronectin Expression in Embryonic Wounds

**Figure 4:**
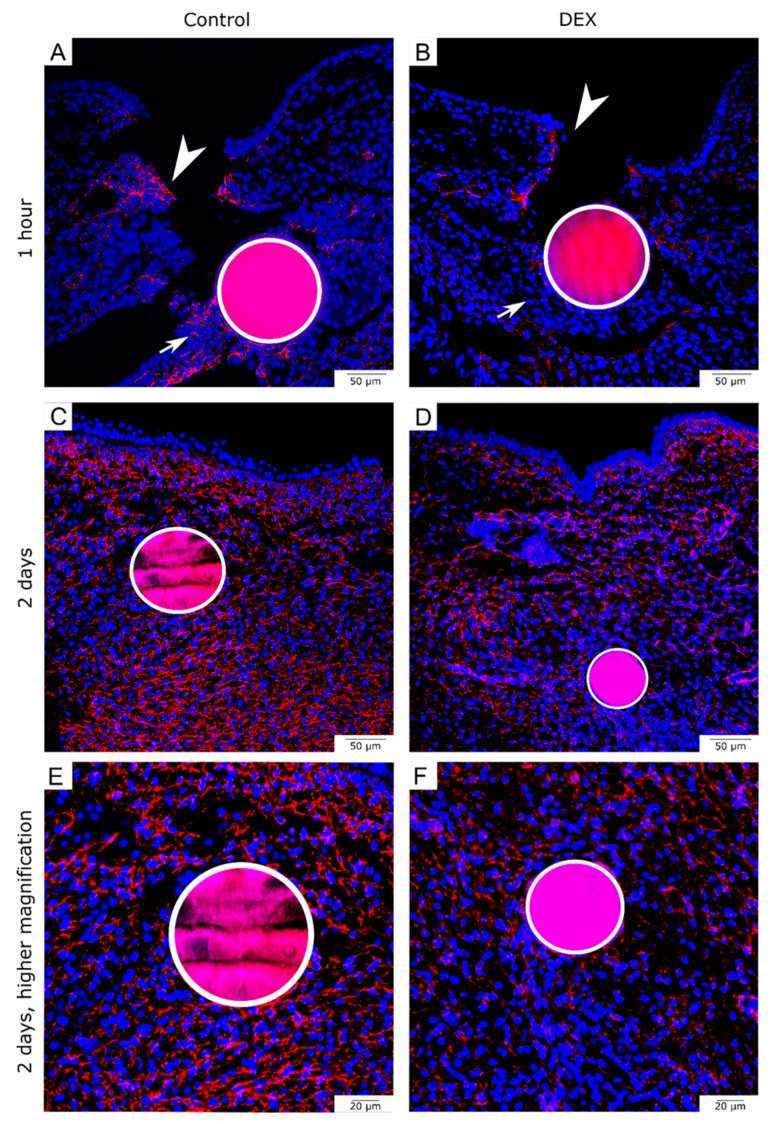
Fibronectin expression in bead-implanted wounds. The images show immunohistochemistry for fibronectin in the bead-implanted wounds. (**A**) shows a control wound after one hour. (**B**) shows a dex wound after one hour. Note the reduced expression of fibronectin at the wound margins (white arrowheads in (**A**,**B**)) and in the tissue surrounding the bead (white arrow in (**A**,**B**)). (**C**) shows a control wound after two days. Note, that Fibronectin is found directly adjacent to the bead. (**D**) shows a dex wound after two days. (**E**,**F**) show the same sections in higher magnification respectively. Note the disturbed matrix pattern of fibronectin in dex wounds after two days, as well as a downregulation of fibronectin expression in the bead area.

For fibronectin expression, we detected the protein by immunohistochemistry at the migrating mesenchymal wound margins of control wounds after one hour (Figure 4A, arrowhead). Moreover, there was fibronectin expression in the immediate area around the control beads (Figure 4A, arrow). The dex wounds showed less fibronectin in the mesenchymal matrix of the wound margins after one hour (Figure 4B, arrowhead). The mesenchymal matrix around the bead also showed less fibronectin expression (Figure 4B, arrow). The reduction of fibronectin expression in dex wounds in comparison to control wounds after one hour was observed in 80% of the experimental group (control: *n* = 4; dex: *n* = 5).

After two days, the subcutaneous mesenchyme of the control wounds heavily expressed fibronectin. The expression pattern in the control wound appeared organized and even (Figure 4C,E). The dex wounds showed a disturbed fibronectin pattern after two days. While there was fibronectin expression in the dermis and around the hematoma, there was less expression in the immediate area around the bead and the matrix appeared disorganized (Figure 4D,F). The reduction of fibronectin expression in dex wounds as well as the disorganized mesenchymal matrix in comparison to control wounds after two days was observed in 100% of the experimental group (control: *n* = 4; dex: *n* = 5).

### 3.4. Local Dexamethasone Administration Temporarily Reduces Mitotic Activity of Embryonic Wound Tissue

**Figure 5:**
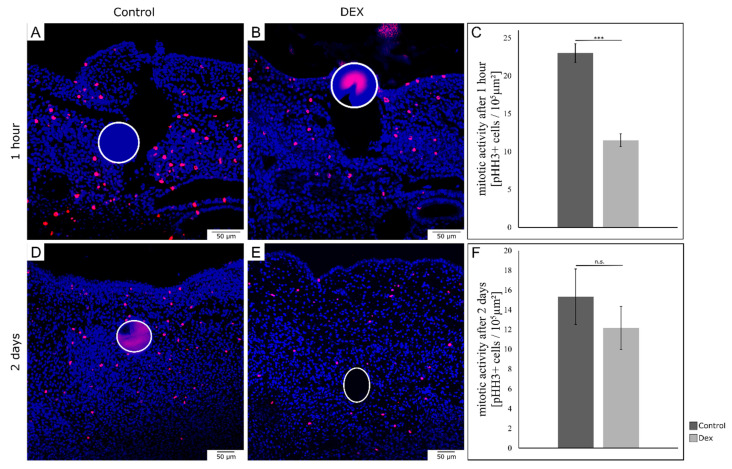
Mitotic activity in bead-implanted wounds. The images show the comparison of mitotic activity in the bead-implanted wound areas using immunohistochemistry for phospho-Histone H3 (pHH3). (**A**) shows a control wound after one hour. (**B**) shows a dex wound after one hour. (**C**) shows the quantification of pHH3-positive cells after one hour. Note the significant reduction in the number of mitotic cells in dex wounds after one hour of wounding (control: *n* = 3; dex: *n* = 3). *** *p* < 0.01. (**D**) shows a control wound after two days. (**E**) shows a dex wound after two days. (**F**) shows the quantification of pHH3-positive cells after two days. After two days, the mitotic activity was insignificantly reduced (control: *n* = 3; dex: *n* = 3). All data are presented as mean ± SE. n.s. = not significant.

In order to analyze mitotic activity during embryonic wound healing after local dex administration, we performed immunohistochemistry for phospho-Histone H3, indicating cell proliferation (Figure 5A,B). After one hour, dex wounds showed a significant decrease in the number of mitotic cells by 50% (*p* = 0.002; control: *n* = 3; dex: *n* = 3) (Figure 5C). After two days, dex wounds showed an insignificant decrease in mitotic activity by 20.6% (*p* = 0.254; control: *n* = 3; dex: *n* = 3) (Figure 5F).

### 3.5. Local Dexamethasone Administration Increases E-Cadherin Expression during Embryonic Wound Healing

**Figure 6:**
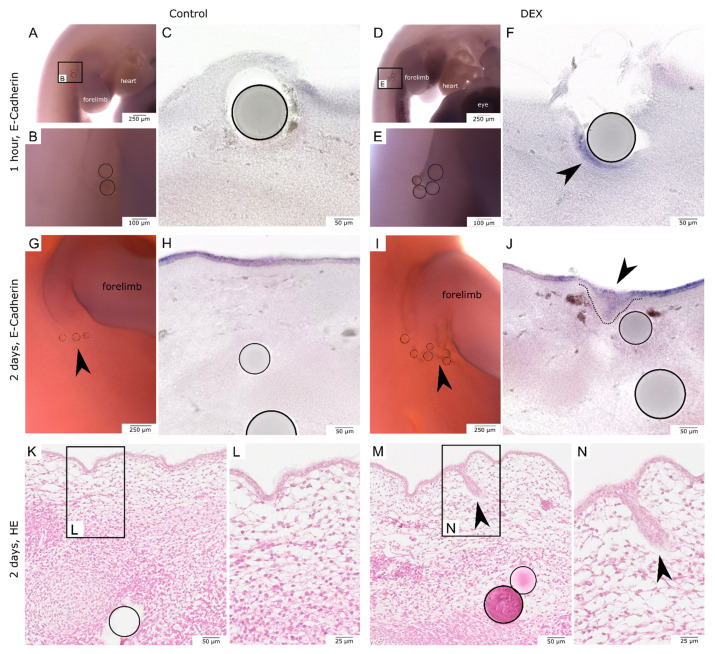
E-Cadherin expression and re-epithelialization of bead-implanted wounds. The images compare the process of re-epithelialization of the wound areas by in-situ hybridization for E-cadherin. (**A**,**B** (higher magnification)) show the whole-mounts of hybridized control wounds after one hour. (**C**) shows a respective vibratome section. (**D**,**E** (higher magnification)) show the whole-mounts of hybridized dex wounds after one hour. (**F**) shows a respective vibratome section. Note the increase in E-cadherin expression in the dex wound (black arrowhead in (**F**)) as well as the open incisional wound. (**G**) shows the whole-mount of a hybridized control wound after two days. (**H**) shows the respective vibratome section. (**I**) shows the whole mount of a hybridized dex wound after two days. (**J**) shows the respective vibratome section. Note that after two days, both wounds have re-epithelialized but the dex wound showed a persisting increase in E-cadherin expression. (**K**–**N**) show the same phenomenon after two days in HE-stained paraffin sections. The epidermal invagination in the HE-stained section of the dex wound is indicated by black arrowheads in (**M**,**N**).

To further investigate the process of re-epithelialization of embryonic wounds after local dex administration, we performed in-situ hybridization for E-cadherin and analyzed vibratome sections. After one hour, the control wounds were almost completely covered with epithelium and showed only little E-cadherin expression (Figure 6A–C). The dex wounds showed an open wound with disturbed re-epithelialization and an increased expression of E-cadherin in the bead-implanted area (Figure 6D–F, arrowhead). This effect was observed in 100% of the experimental group (control: *n* = 5; dex: *n* = 5).

After two days, the increase of E-cadherin expression in dex wounds persisted. While the control wounds had healed and evenly expressed E-cadherin in the epidermis (Figure 6G,H), the dex wounds showed an increase in E-Cadherin expression. The whole mounts of dex wounds after two days showed more E-cadherin expression in the bead-implanted area (Figure 6I, arrowhead). The vibratome sections revealed a thickening of the E-cadherin positive epidermal cells extending into the subcutaneous mesenchyme (Figure 6J, arrowhead). The increase in E-cadherin expression in the dex wound area in comparison to the control wounds was observed in 100% of the experimental group (control: *n* = 5; dex: *n* = 5). The epithelial thickening presented as an epidermal invagination in paraffin sections of dex wounds (Figure 6K–N, arrowheads). 

### 3.6. Local Dexamethasone Administration Temporarily Disturbs Expression of Dermo-1

**Figure 7:**
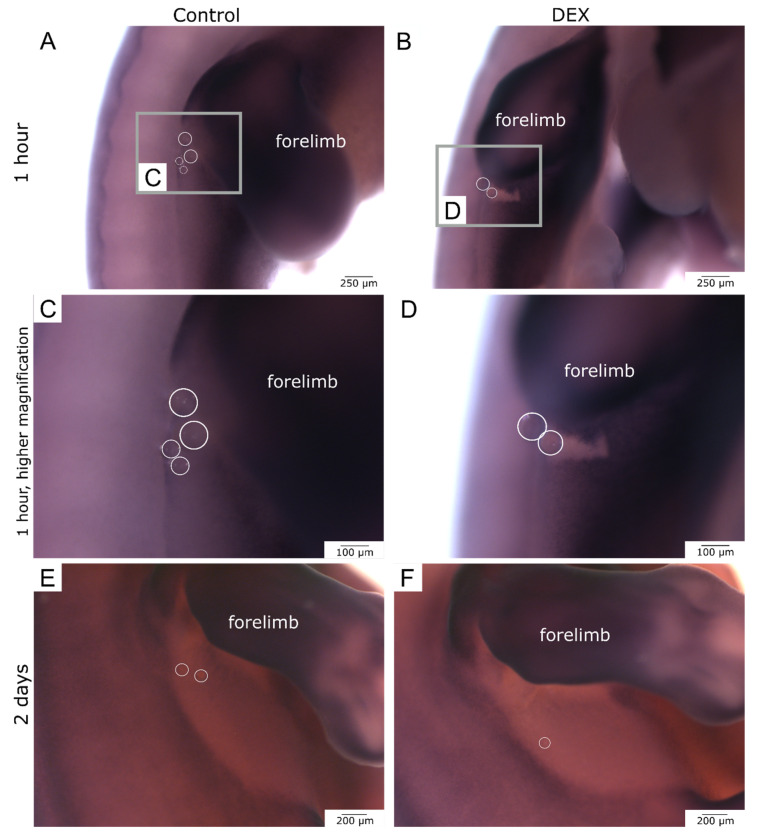
Dermo-1 expression in bead-implanted wounds. The images display the expression of Dermo-1 in the bead-implanted wounds. (**A**) shows a whole- mount of a hybridized control wound after one hour. (**C**) shows the same wound in higher magnification. (**B**) shows a whole mount of a hybridized dex wound after one hour. (**D**) shows the same wound in higher magnification. Note that in the dex wound the Dermo-1 expression pattern showed a bigger disruption after one hour. (**E**) shows a whole-mount of a hybridized control wound after two days. (**F**) shows the respective dex wound. Note that both wounds have restored a proper Dermo-1 expression pattern after two days.

In order to predict further dermis establishment after skin wounding during embryonic development, we performed in-situ hybridization for Dermo-1. The expression pattern of Dermo-1 was disrupted by wounding the skin. After one hour, the control wounds showed a further progressed regeneration of the Dermo-1 expression pattern in the bead-implanted area in comparison to the dex wounds (Figure 7A–D). This effect was observed in 100% of the experimental group (control: *n* = 5; dex: *n* = 5). After two days, the control- and dex wounds had both regenerated the Dermo-1 expression pattern (Figure 7E,F). This phenomenon was also visible in 100% of the experimental group (control: *n* = 5; dex: *n* = 5).

## 4. Discussion

The aim of the current study was to investigate the effects of local dexamethasone (dex) administration on the complex process of embryonic wound healing of skin incisional wounds in developing chicken embryos. By performing standard histological techniques, immunohistochemistry, and in-situ hybridization we analyzed skin regeneration one hour and two days after in-ovo implantation of dex- or control beads into skin wounds. 

In general, we detected an impaired process of embryonic wound healing after local dex administration, shown by delayed wound closure, presumably due to impaired migration and proliferation of cells and disturbed re-epithelialization, resulting in mesenchymal tissue defects as well as epidermal alterations after two days. 

In line with our findings for embryonic wound healing, glucocorticoids are known to impair the healing process of adult wounds in various ways, for example by delaying the proliferation and migration of keratinocytes as well as inhibiting epithelialization [38,39]. 

The regulation of keratinocyte differentiation and fibroblast proliferation during adult wound healing is known to be regulated by the intermediate filament protein vimentin via TGF- β1 [40]. During embryonic wound healing, vimentin-deficient mouse embryos presented with impaired wound healing due to “failure of mesenchymal contraction” [14]. Our observation of a reduced vimentin expression in dex wounds is in line with these findings. Since glucocorticoids are known to reduce vimentin expression by inhibiting TGF- β1 induced epithelial-to-mesenchymal (EMT) transition in other tissues [41,42], one can hypothesize that this particular pathway contributed to the deceleration of mesenchymal contraction in embryonic skin wounds. 

As a consequence thereof, the upregulation of the epithelial marker E-cadherin observed in dex wounds can also be associated with the signaling pathways involved in EMT [41,42]. Our demonstration of dex-induced upregulation of E-cadherin during impaired wound healing is in line with findings in *Drosophila melanogaster* embryos, which demonstrated that E-cadherin removal from the wound site is a prerequisite for proper healing [15]. Overexpression of E-cadherin in embryonic wounds was described to lead to slower wound closure and insufficient actomyosin cable formation [15]. 

For fibronectin expression, we found a decrease in dex wounds after one hour, especially in the migrating wound margins. While the effects of glucocorticoids on fibronectin synthesis are discussed contradictorily [43,44], the decrease of fibronectin expression after dex administration in embryonic wounds is consistent with the morphological changes seen as well as the described markers and pathways of EMT [41]. The reduction of fibronectin expression persisted after two days in the dex bead-implanted area and the surrounding mesenchymal matrix appeared disorganized in comparison to the control wounds. Fibronectin is a glycoprotein of the extracellular matrix regulating cell adhesion, cell migration, cell proliferation, and matrix assembly [45]. Since fibronectin has been detected in adult as well as fetal wounds [46], one can expect it to have a key role during the embryonic regeneration process. Our findings of reduced fibronectin expression could be a contributing factor to the delayed process of mesenchymal contraction and re-epithelialization during embryonic wound healing, as it has been described for adult wound healing or in vitro [47,48,49]. Moreover, the lack of organized matrix formation in dex wounds after two days reflects characteristics of scarring or fibrosis seen in adult wounds [46]. 

Adult wounds are known for the proliferative phase, including increased mitosis of keratinocytes, fibroblasts, and other cells, necessary for the formation of granulation tissue and restorage of the epithelium [50]. For embryonic wound healing, it has been discussed whether cell proliferation aids wound closure, how fast it can be upregulated, and to what extent it is even necessary during embryonic tissue repair [10]. Nevertheless, we observed a significant decrease in the number of mitotic cells one hour after local dex administration. A limitation of this result is, that it was not determined clearly, whether the rather fast decrease in the number of mitotic cells is due to dex-induced downregulation of the proliferation rate, due to cell cycle arrest, or due to cell death. All described possible phenomena have been associated with glucocorticoid exposure [51,52]. After two days of wound healing, the number of mitotic cells was still reduced in dex wounds but to a lesser extent. The decrease in presumable cell proliferation of dex wounds possibly contributed to the decelerated regeneration process and the significant decrease in cell density of the subcutaneous mesenchyme.

For further post-wounding development of the skin after local dex administration, we observed that, the expression pattern of dermal marker Dermo-1 was temporarily disrupted due to the mechanical effects of the incisional wound. Accompanied by the impairment of mesenchymal contraction and re-epithelialization in dex wounds, the reconstitution of a regular Dermo-1 expression pattern was potentially decelerated in dex wounds. After two days the regeneration capacities of the chicken embryo in the particular developmental stage allowed for a reconstitution of the regular Dermo-1 expression pattern in the control as well as in the dex wounds, presumably indicating only a temporarily prolonged disruption of the Dermo-1 expression pattern by local glucocorticoid administration, without long-lasting effects on dermis development. Since Dermo-1 plays an important role in establishing dense, thick dermis and inducing skin appendage formation [16,53], one can hypothesize that despite the impaired process of wound healing caused by local dex administration, the embryonic wounds are able to recover with skin appendage formation. 

To conclude, our study provides first insights into the local effects of dexamethasone on embryonic wound healing. By impairing mesenchymal contraction, re-epithelialization, and various other cellular processes, the healing of the skin incisional wounds was decelerated by the glucocorticoid resulting in morphological changes in the skin, such as increased epithelialization and disorganized matrix formation, which could reflect some particular traits of fibrotic skin regeneration seen in adults. It needs to be examined in more detail, if the local dexamethasone administration leads to long-term consequences, but our results propose that despite the mesenchymal defects, the embryonic skin has the potential to restore its integrity completely, antagonizing the notion of dex-induced characteristics of fibrosis. Moreover, the study performed in the chicken embryo provides a useful technique for local embryonic drug administration in an in-vivo model, allowing for a relatively simple investigation of drug-induced effects due to its ex-utero accessibility and continuous visuality. 

The results gained in the chicken embryo can contribute to a better understanding of scarless embryonic wound healing and how prenatal stress hormones or the therapeutic administration of synthetic glucocorticoids might interfere with the underlying molecular processes, possibly indicating that glucocorticoid therapies in prenatal clinical practice should be carefully evaluated. 

## Data Availability

Data is contained within the article.
